# The multi-subject cooperation mechanism of home care for the disabled elderly in Beijing: a qualitative research

**DOI:** 10.1186/s12875-022-01777-w

**Published:** 2022-07-26

**Authors:** Ruyi Zhang, Zhiying Zhang, Yingchun Peng, Shaoqi Zhai, Jiaojiao Zhou, Jingjing Chen

**Affiliations:** 1 School of Medical Humanities, Capital Medical University, No. 10, Xitoutiao, You An Men Wai, Fengtai District, Beijing, 100069 China; 2 Fengtai District, Xiluoyuan Community Health Service Center, Beijing, 100077 China; 3 Huairou District, Liulimiao Community Health Service Center, Beijing, 101400 China

**Keywords:** Disability, Elderly, Multiple subjects, Home care, Qualitative research

## Abstract

**Background:**

Currently, population aging has been an obstacle and the spotlight for all countries. Compared with developed countries, problems caused by China’s aging population are more prominent. Beijing, as a typical example, is characterized by advanced age and high disability rate, making this capital city scramble to take control of this severe problem. The main types of care for the disabled elderly are classified as home care, institutional care, and community care. With the obvious shortage of senior care institutions, most disabled elderly people are prone to choose home care. This kind of elderly care model is in line with the traditional Chinese concept and it can save the social cost of the disabled elderly to the greatest extent. However, home care for the disabled elderly is facing bumps from the whole society, such as lack of professional medical care, social support and humanistic care, and the care resources provided by a single subject cannot meet the needs of the disabled elderly.

**Objective:**

Based on the demands of the disabled elderly and their families, this study aims to explore the current status of home care service, look for what kind of care is more suitable for the disabled elderly, and try to find an appropriate elderly care mechanism which could meet the diverse needs of the disabled elderly.

**Methods:**

A total of 118 disabled elderly people and their primary caregivers were selected from four districts of Beijing by using multi-stage stratified proportional sampling method. A one-to-one and semi-structured in-depth qualitative interview were conducted in the study to find out the health status of the disabled elderly, the relationship between the disabled elderly and their primary caregivers, and utilization of elderly care resources, etc. The views of the interviewees were analyzed through the thematic framework method. All the methods were carried out in accordance with relevant guidelines and regulations.

**Results:**

The results showed that the average age of 118 disabled elderly is 81.38 ± 9.82 years; 86 (72.9%) are severe disability; 105 (89.0%)are plagued by chronic diseases; the average duration of disability is 5.63 ± 5.25 years; most of disabled elderly have 2 children, but the primary caregiver are their own partner (42, 35.6%), and there is an uneven sharing of responsibilities among the disabled elderly's offspring in the process of home care. The disabled elderly enjoy medical care services, rehabilitation training, daily health care, psychological and other demands. However, the disabled elderly and their families in Beijing face a significant financial burden, as well as physical and psychological issues. The care services provided by the government, family doctors, family members and social organizations fall far short of satisfying the diverse care needs of the disabled elderly.

**Conclusions:**

In order to effectively provide home care services for the disabled elderly, it is therefore necessary to establish a coordination mechanism of multiple subjects and give full play to the responsibilities of each subject. This study proposes a strengthening path for the common cooperation of multiple subjects, which taking specific responsibilities and participating in the home care for the disabled elderly: (1) The government should give full play to the top-level leading responsibilities and effectively implement people-oriented measures to the disabled elderly. (2) Family doctors strengthen their responsibilities as health gatekeepers and promote continuous health management of the disabled elderly. (3) Family members assume the main responsibility and provide a full range of basic care services. (4) Social forces promote supplementary responsibilities of public welfare and expand the connotation of personalized care services. (5) The disabled elderly should shoulder appropriate personal responsibility and actively cooperate with other subjects.

## Introduction

With the deepening of population aging, there are a growing number of the elderly, which has become a social problem cannot be ignored. According to statistics, Beijing is one of the most aging cities in China. The total permanent population of 60 years old and above in 2020 has reached 4.299 million, and the 65 years old and above population in 2020 is 2.912 million. Compared with 2016, the proportion of population over 60 years old and above has risen from 15.5% to 19.6%, the proportion of population over 65 years old and above increased from 10.3% to 13.3% [1]. The aging population in Beijing presents the distribution characteristics of a high degree of aging in the urban areas and relatively low in the suburbs, and a trend of the rapid growth of advanced age. Moreover, the problem of population aging has caused the growing number of the disabled elderly. With the average life expectancy of the elderly has risen year by year, it is inevitable that the disability life expectancy has simultaneously increased. The disabled elderly will be the most severely affected group, because the advanced age and disability are the main influential factors which restricted their lives [2].

In 2020, there were 130,000 disabled elderly people in Beijing who had obtained the Civil Affairs Department’s disability certification and received relevant subsidies. With the continuous expansion of the number of the disabled elderly, how to better meet their various needs has become an urgent social problem to be solved. According to relevant study, the needs of the disabled elderly mainly lies in three aspects, including daily living care, emotional support and medical services [3]. The main types of care for the disabled elderly are divided as home care, institutional care, and community care. For the obvious shortage of the elderly care institutions, most disabled elderly people choose home care. This kind of elderly care model is in line with the traditional Chinese concept and it can save the social cost of the disabled elderly to the greatest extent [4]. However, home care for the disabled elderly except for the basic maintenance of daily living care, there are serious deficiencies in the supply of care services for the disabled elderly, such as medical care, rehabilitation training, spiritual comfort, cultural leisure and other services [5]. Family members are short of professional nursing knowledge and care tools, and they are unable to employ medical emergency treatment for the disabled elderly.

In order to deeply understand what difficulties that disabled elderly are being confronted with and what support is required in the process of home care, this study selected disabled elderly and their families with different characteristics from urban areas, mountainous areas, and plains of Beijing for in-depth interviews. The approach is designed to establish a home care service system and enhance the quality of care services for the disabled elderly.

## Interviewee and methods

### Interviewee

#### Selection of interviewee

Taking the level of economic development and the linear distance from Tiananmen Square of each district into account, this study has selected 15 community health service centers (CHS centers) in 4 districts of Beijing as research sites, 2 from urban areas and 2 suburbs respectively (namely Xicheng District, Fengtai District, Daxing District, and Huairou District). Then, 3–4 CHS centers are selected from each district. Xicheng and Fengtai District are the core functional areas in Beijing, in terms of the 2020 Beijing Regional Statistics Yearbook [6], Xicheng’s per capita disposable income is the highest among the six urban areas, at 88,291 yuan; while Fengtai has the lowest per capita disposable income, at 65,215 yuan. Daxing District is a representative of the plain areas in the suburbs of Beijing. In 2020, the per capita disposable income of Daxing is 47,432 yuan, which is the 9^th^ place among the 16 districts in Beijing. Huairou District is a representative of mountainous areas in the suburbs of Beijing, its per capita disposable income is 40,067yuan, ranking 12^th^ among all the districts in Beijing.

Now, the CHS centers in Beijing all have family doctor teams. Policies detail the tasks to respond to care for disabled elderly, family doctors should provide contracted services and conduct health management for the group. Based on the situation of the disabled elderly (age ≥ 60) who have contracted by the family doctor team in each CHS center, 118 cases of disabled elderly are selected according to the ratio of 3% from the health management files by adopting multi-stage stratified proportional sampling method, the 118 disabled elderly and their family members/caregivers are interviewed personally and in-depth during the same period.

According to the Guest's(2020) in qualitative research [7], the criterion for assessing data saturation is when the amount of new information obtained from 2 new respondents is less than 5% of the total information obtained from the previous 4 interviewees. The sample size of 118 disabled elderly has reached maximum data saturation of this research.

### Inclusion criteria and exclusion criteria

Inclusion criteria: 1)The disabled elderly who was assessed by the Beijing Municipal Civil Affairs Department(The assessment of the disabled elderly firstly is applied by the elderly or their family members, then assessed by professionals from healthcare institutions according to the “Implementation Measures for Comprehensive Assessment of the Ability of the Elderly in Beijing”. The assessment focuses on the elderly who are unable to take care of themselves due to injury, illness, disability, infirmity, etc., as well as those people who have a clear medical diagnosis and they are still unable to take care of themselves after more than six months of treatment.); 2)The disabled elderly are able to express their personal wishes independently and clearly; 3)Family members, who have provided long-term care (more than six months) for the disabled elderly, are accompanied to participate in this study and they also have obtained informed consent.

Exclusion criteria: 1)The disabled elderly or their caregivers have a mental disorder (Due to communication barriers and low cooperation of participants with mental disorders, it is difficult for them to complete one-to-one interviews.); 2)The disabled elderly are unwilling to participate or not able to cooperate with the research; 3)Caregivers are unwilling to accompany the disabled elderly to participate in this study.

### Method of research: Qualitative research

Under the national and Beijing’s relevant policies of home care for the disabled elderly, the research team has considered the humanistic environment, regional characteristics and the actual situation of the contracted services in Beijing, compiled an interview outline after extensive review of relevant literature and repeated discussion by panel experts.

The content of the interview contains the basic information of disabled elderly, their health status, the status quo of geriatric care, the demands of the disabled elderly and their families, the degree of utilization of home care resources, and specific advice for the disabled elderly of home care. One-to-one, semi-structured in-depth personal interviews were conducted in this study. All the interviewers were trained by a unified standard in advance to avoid inductive questions and reduce research bias. Before the interview, the interviewer gave a detailed introduction of the study purpose, methods, content and confidentiality principles to the interviewees and obtained informed consent [8]. In order to reduce traffic inconvenience of the disabled elderly, the interviews were engaged in their homes, to ensure the accuracy and completeness of the information, and the entire processes recorded with the consent of the interviewees. The content of the recordings was transcribed in detail by members of the research team within 24 h after the interview completed to ensure the authenticity of the interview content. The interviewees were anonymized, and the disabled elderly's names were coded from N1 to ~ N118.

### Method of analysis: Thematic framework

The content analysis method is used to conduct qualitative analysis of the interview data, and the data are classified and analyzed by identifying themes, data labeling, and extracting core information [9]. The analysis process includes four steps [10]: 1)Establishing a general impression of all the content from the disabled elderly and their families with an open mind, looking for preliminary themes related to the home care for the disabled elderly. 2)Identifying the meaning units from themes, coding the relevant content related to the subject. 3)Sorting and arranging themes into categories by code frequency, then sorting them into subcategories. 4)Putting the pieces together to reconceptualize the content. By synthesizing the contents from subcategories, the description and concepts develop as an analytical text supported by citations, which can illustrate the research theme or finding.

## Results

Using thematic framework methods, the content of the interview is summarized into six themes as follows:

(i) The reasons for caregiver selection and the relationship between the disabled elderly and their primary caregivers;
(ii) The difficulties faced by family caregivers in providing home care for the disabled elderly;
(iii) The willingness of the disabled elderly and their families to receive volunteer services;
(iv) The content of care services provided by family doctors;
(v) The current status of care services provided by neighborhood committees, volunteers, neighbors, and care institutions;
(vi) What the government, family doctors, society, family members and the disabled elderly themselves should do to improve the quality of home care.

### Basic information

#### Demographic characteristics of the disabled elderly

The demographic characteristics of the 118 disabled elderly people interviewed is shown in the Table 1. (1) Urban–rural distribution: There are 63 disabled elderly people from cities and the rest (55) are from rural areas. (2) Gender: There is a balanced distribution between men and women, each accounting for 50%. (3) Age: Among the participants, the youngest is 61 years old and the oldest is 99 years old, with an average age of 81.38 ± 9.82 years old. The seniors over 80 years old have exceeded half of the interviewed. (4) Education level: 41.5% of the interviewees have a primary school education or below. (5) Disability level: The majority of the elderly are severely disability, with 86 people, accounting for 72.9%. (6) Duration of disability: The minimum disability period is within 1 year, and the maximum period of disability is 30 years. The average duration of disability is 5.63 ± 5.25 years. The majority with disability duration are within the range from 1 to 10 years. (7) Morbidity of chronic diseases: Only 13 disabled elderly people interviewed are in good health condition, 75 (63.6%) are subjected to hypertension, and 42 (35.6%) are type 2 diabetes. (8) Basic medical insurance: Of the 76 persons (64.4%) have medical insurance as city and rural residents, 36 (30.5%) persons have medical insurance as city employees, 5 (4.2%) persons have public medical care, and 1 (0.8%) person has low-cost medical aid.

**Table 1:** Demographic characteristics of the disabled elderly

Items	Number of interviewees (%)
**Urban–rural distribution**	
Rural area	55 (46.6)
Urban area	63 (53.4)
**Gender**	
Male	59 (50.0)
Female	59 (50.0)
**Age**	
60 ~	18 (15.3)
70 ~	26 (22.0)
80 ~	44 (37.3)
90 ~	30 (25.4)
**Education**	
Elementary school or below	49 (41.5)
Secondary school	44 (37.3)
Junior college	18 (15.3)
University or above	7 (5.9)
**Disability level**	
Mild disability	12 (10.2)
Moderate disability	20 (16.9)
Severe disability	86 (72.9)
**Duration of disability(year)**	
< 1	24 (20.3)
1 ~	79 (66.9)
10 ~	15 (12.7)
**Morbidity of chronic diseases**	
Not have	13 (11.0)
Have	105 (89.0)
**Basic medical insurance**	
Medical insurance for urban and rural residents	76 (64.4)
Urban employee medical insurance	36 (30.5)
Low-budget medical assistance	1 (0.8)
Public medical	5 (4.2)
**Total**	118 (100)

#### The main reasons resulting in the disability

As shown in the Table 2, the main reasons resulting in the disability are diseases, such as hypertension, diabetes, rheumatoid arthritis, lumbar spine stenosis and other diseases prevalent in the elderly. Cerebral infarction is the main causes of disability among the disabled elderly, followed by malignant tumors and myocardial infarction. Due to advanced age and external force injuries, the number of the disabled elderly are accounted for 11(9.3%) and 12(10.2%), respectively.

**Table 2:** The main reasons resulting in the disability

The main reasons	Number of interviewees(%)
Old age	11 (9.3)
Cerebral infarction	36 (30.5)
Myocardial infarction	7 (5.9)
Malignant tumor	9 (7.6)
Other diseases	43 (36.4)
External force damage	12 (10.2)
Total	118 (100.0)

### The current situation of home care for the disabled elderly

#### The primary caregiver of the disabled elderly

Of the 118 disabled elderly interviewed, the 4 have no children, 22 of them have only one child, and the majority have two children (33.9%). As shown in Table 3, the primary caregivers of the disabled elderly are mostly their partners, which accounts for 35.6% (42). It is noteworthy that 68 family caregivers mentioned that they had heavy psychological stress, 36 of them are the partners of the disabled elderly.

**Table 3:** The primary caregiver of the disabled elderly in home

Primary caregiver	Number of interviewees(%)
Wife or husband	42 (35.6)
Son	18 (15.3)
Daughter	12 (10.2)
Daughter-in-law	13 (11.0)
Son-in-law	1 (0.8)
Nanny	21 (17.8)
Other relatives	5 (4.2)
No fixed caregiver (children’s shift)	6 (5.1)
Total	118 (100.0)

#### The reasons for caregiver selection and the relationship between the disabled elderly and their primary caregivers

It is found that most of primary caregivers are their wives or husbands (42/118). In the interview, the disabled elderly indicated that the main reason for choosing their partners as caregivers below:

(1) Their children are busy at work and only their partners can take care of them (31/118).

As the disabled elderly N16 mentioned, “My son and daughter have not retired yet, they are busy reconciling work and family responsibilities, they also need to take care of their grandchildren and deal with their own housework.”

(2) It is more comfortable to live with their partners (11/118).

The interview results also demonstrates that most of disabled elderly choose to live in their son’s home and looked after by their sons and daughters-in-law, or a nanny (37/118), while only a few elderly choose to live in their daughter’s home. The reasons are as follows:

1) They think their sons should be held responsibility (7/118).

2)They think their married daughter belongs to other family and they don’t want to bother them.

The disabled elderly N23 mentioned, “In our country, the daughter who married is like the water poured out. I don’t want to trouble my daughter anymore because she has her own family.”

#### The division of responsibilities among the family members

With regard to the division of care responsibilities among family members, the disabled elderly interviewed generally stated that there is no clear division of responsibilities (97/118).

Only a few families have a clear division of responsibilities for taking care of the disabled elderly (21/118), they share the responsibilities by using the following strategies:

(1) The offspring of the disabled elderly allocate economic responsibilities and care responsibilities (15/118).

The disabled elderly N51 mentioned that “I have many children, but they all have their own business to cope with. The way to solve the problem of care responsibilities is that the second daughter and the youngest daughter pay money monthly for me and the eldest daughter who is mainly responsible for caring.”

(2) The disabled elderly take turns to live in their children's home (6/118).

The disabled elderly N91 mentioned, “I have 4 children, and I live in each child’s home for 3 months in a year. Every child can observe his/her filial piety.”

#### The disabled elderly choose to use the same caregiver steadily for a long time or change regularly

(1) The disabled elderly use the same caregiver steadily for a long time (100/118). The main reasons are as follows:

1) The caregiver who take a long term care for the disabled elderly is more familiar with the health status of the elderly, which is beneficial to their health (21/118).

For example, the disabled elderly N85 said, “I think it is good for one person to take care of me for a long time, because regularly change the caregiver is not good for my health.”

2) The emotional demands (19/118).

As the disabled elderly N50 mentioned, “I also hope not to change the person who attends on me for a long time, because I will feel very close to someone who has lived with me for a long time.”

3) The disabled elderly need to adapt the caregiver’s living habits (10/118).

For example, the disabled elderly N20 stated, “Everyone has their own living habits, it is difficult for me to adapt to other’s lifestyle. If I change the caregiver, I still need a long period of adaptation.”

4) For some families, there is no suitable person to replace, and only one person can undertake such task (60/118).

For example, the daughter-in-law of the disabled elderly N43 mentioned, “The strong hope of my family is to maintain the status quo. The mother-in-law only knows me now. If others touch her, she will get angry.”

(2) The disabled elderly change the caregivers regularly (18/118). The main reasons are as follows:

1) The sole caregiver is under a lot of pressure (7/118).

As mentioned by N64, a disabled elderly, “I think it’s better to rotate regularly, because there is too much pressure for my children to take care of me.”

2) The offspring share the care responsibilities equally, which is relatively fair (9/118).

For instance, the disabled elderly N36 mentioned, “There is no dispute over the care responsibilities of multiple-child family to share the responsibilities equally, and it is more beneficial for children to look after me.”

3) Nanny has highly mobility (2/118).

For example, the disabled elderly N67 said that “The nanny needs to be replaced frequently because she must go home. She also has her own family, and I cannot force her to stay my house for a long time.”

### The difficulties that the primary caregiver may face in the process of home care (answered mainly by the family members of the disabled elderly, the disabled elderly supplemented on the site)

(1) Caring for the elderly requires a lot of effort, and the caregiver’s personal health generally is too weak to take on the heavy pressure (92/118).

For example, the disabled elderly N3 mentioned: “The main problem now is that my wife is too old to care me thoughtfully and meticulously. Because I am handicapped and it’s hard for her to move my body. Moreover, my epilepsy will get sick from time to time. When the situation is critical, I still need my children to accompany me to the hospital.”

(2) The physical health of the disabled elderly needs a lot of drugs to maintain, but the financial abilities of their families are limited (83/118).

As the disabled elderly N117 indicated, “I’m just a farmer and I don’t have any income. My sons and daughters-in-law are both disabled currently, they are unable to work and they also have no financial ability to bear my extra expenses.”

(3) Being with the disabled elderly for a long time, the psychological pressure of the primary caregiver is heavy(68/118).

For example, the son of the disabled elderly N33 said, “As a caregiver, my biggest difficulty is that I am occupied in looking after my disabled parents. Moreover, the emotional status of my parents are unstable, they sometimes act like children. It requires me to be very meticulous, considerate, and obedient to them. I often wipe my tears off secretly, and I feel too much pressure to talk about it.”

(4) There is a gender gap between the caregiver and the disabled elderly(e.g. daughter-in-law takes care of father-in-law), and the nursing process is embarrassing (61/118).

An elderly female N51 mentioned, “I am getting older and I cannot take care of myself. It is not proper for my son to be. my helping hand, such as bathing, so I have to wait for my daughter when she is free.”

(5) It is difficult to keep the balance of caring for the elderly with their own works and their family lives (53/118).

For example, N59’s son said, “My wife had a car accident a few years ago, and the steel plate is still in her body. But I also have to work, it is stressful for me to look after my mother and my wife at the same time.”

(6) The primary caregivers lack of professional nursing knowledge (34/118).

As the daughter of N96 mentioned, “My father stayed in bed for a long time, I have to perform tasks such as ‘nasal feeding’ and 'sputum suction' for the elderly. It’s difficult for me to conduct it, and I don’t know how to do.”

(7) There is a controversial issue about the division of responsibilities within the disabled elderly family (12/118).

For example, the young daughter-in-law of N107 as the primary caregiver said, “The elderly has 6 children, and I am the daughter-in-law of the youngest son. I have said many times that children should discuss the elderly’s care issues together, but it is difficult to organize family meetings. Some children rarely pay little money for the elderly, and even some children neither pay nor contribute. I am 53 years old and I also should take care of my parents, but I have spent much time on this family.”

#### The situation of care services provided by neighborhood committees, volunteers, neighbors, care institutions, etc*.*

(1) No care services provided (63/118)

The disabled elderly N19 mentioned, “Currently, the neighborhood committee or other institutions haven’t provided any care services for us. Although the neighborhood committee in our village has an old-age care institution, it is only for the uninsured or those who have no children.”

The disabled elderly N60 told the researcher, “When I lived in my hometown, my neighbors knew each other, but now I have moved to a new community, no one comes to help me, even greeting. Everyone in the building closes the door and avoids contacting with others.”

(2) Assistance mainly from the neighborhood or village committee (55/118)

The disabled elderly N1 said that “The brigade members of our village will come to my home to deliver some free condolences to me during the Spring Festival or other holidays, and organize people to give us free haircuts every two months.”

(3) Help from neighbors or volunteers (9/118).

The disabled elderly N65 recalled to mind, “Volunteers have come to my home and know some essentials, but they come occasionally and the activities are not permanent. They can’t help me if there is an emergency.”

### The current status of contracted services provided by family doctors for the disabled elderly

The 118 disabled elderly interviewed have their own contracted family doctors from the local CHS center and they all know the doctors’ telephone number. Among these participants, only the 2 said that they had not received any services from the family doctors, while the 116 disabled elderly were provided different services by their family doctors. The order of service items are as follow: regular telephone follow-up, health knowledge guidance, regular physical examination, changing of urinary catheter and gastric tube at home, injection at home, WeChat or telephone consultation at any time, making appointments or referrals, rehabilitation training, and psychological comfort.

(1) Regular telephone follow-up (101/118)

The disabled elderly N88 mentioned, “My family doctor call me regularly to ask about my health condition and provide me with medication guidance to understand my health better.”

(2) Health knowledge guidance (98/118)

The caregiver (wife) of the disabled elderly N91 mentioned that “Family doctors will come to the village to hold a health lecture and teach us how to take care of the disabled, such as how to prevent bedsores, I think what I have learned is good for my husband.”.

(3) Regular physical examination (84/118)

The disabled elderly N7 mentioned that “Family doctors will provide door-to-door services, such as ECG examination, blood pressure and blood glucose measurement, etc.”

(4) Changing the urinary or gastric tube and deliver medicine at home (78/118)

As the wife of the disabled elderly N22 mentioned, “Family doctor changing the urinary catheter and gastric tube at home has helped me solve a big problem, because I am too old to take my husband to the hospital regularly.”

(5) At-home injection (69/118)

The disabled elderly N8 mentioned, “Now I have to get two injections every week. I can’t go to the hospital by my own because of the disability or call an ambulance every time as a result of my poor financial ability, so it is a wise choice that call my family doctor to come and inject at home.”

(6) Telephone or WeChat consultation at any time (65/118)

The disabled elderly N13 mentioned, “Owing to geographical location, it’s difficult for us who live in the mountainous area to see a doctor. Therefore, many of health problems have been solved through WeChat online.”

(7) Appointments or referrals (43/118)

The disabled elderly N64 referred to, “My family doctor can help me make appointments and register in a large hospital, so that I can go to the superior hospital conveniently.”

(8) Rehabilitation training (34/118)

The disabled elderly N55 mentioned, “The family doctor has helped me with rehabilitation training, so I am able to walk with the railing now.”

(9) Psychological comfort (33/118)

The disabled elderly N83 mentioned, “For those people who are seriously ill, most doctors would pay more attention to their physical health. While my family doctor will chat with me every time, which makes me feel I have a doctor friend.”

### The service demands of the disabled elderly and their families for the multiple subjects which involved in the process of home care

By using the thematic framework methods, we summarized the service demands of the disabled elderly and their families for the multiple subjects which involved in the process of home care, the analysis process of the different subjects from the interviews are shown in Table 4.

**Table 4:** The analysis process of the different subjects from the interviews

Subject	Code	Category	Subcategory
Government	G1	Subsidy	Financial support, allowance, money
	G2	Service	Volunteer service, mutual aid services
	G3	Standard	Service content, service standards
	G4	Policy	Disability assessment, laws, regulations
	G5	Consideration	Care, spiritual concern
Family Doctor	D1	Basic service	Medical service scope, basic medical care
	D2	Education	Health education, health messages
	D3	Personal service	Family hospital beds, door-to-door service
	D4	Concern	Care, concern, release stress
	D5	Convenience	Green channel, convenience
Family Member	M1	Financial support	Financial support, money, economic
	M2	Company	Care, company, psychological concern
	M3	Patience	Patience, careful, kind
	M4	Skill	Knowledge, professional skill
Society	S1	Free service	Voluntary service, free service
	S2	Respect	Harmony, respect, approve
	S3	Paid service	Professional platform, nanny
	S4	Community help	Neighborhood help, community mutual aid, committee care
	S5	Financial aid	Financial, money
The Elderly	E1	Disability	Incapable, no responsibility
	E2	Behavior	Receive advice, adopt behaviors
	E3	Attitude	Kind, nice, temper less

#### Government

(1) Increasing financial support (91/118)

The disabled elderly N9 mentioned, “For our peasant family, having a disabled elderly means severe financial pressure. I hope that the government will provide financial assistance for us every month. I think the disability subsidy of 600 RMB is not enough to meet life’s basics.”

(2) Strengthening efforts to encourage volunteer service (88/118)

The disabled elderly N28 mentioned that “Through our government’s publicity on volunteer service, more young people would like to participate in the volunteer activities and help more people like us.”

(3) Expanding the service scope of the elderly care post, increasing the service items, and reducing the charging standard (84/118)

The disabled elderly N81 mentioned, “I think the service items of the elderly care post are limited and the disability subsidy can only be used in the post, while I believe the service charges of the post are more expensive than the outside.”

(4) Improving relevant policies, laws and regulations for the elderly (68/118)

The disabled elderly N92 said, “I hope to accelerate the process of assessing the qualification. At the same time, the subsidy should be made a clearer policy, and establish a mechanism for supervision and feedback.”

(5) Caring more for the elderly group (79/118)

The disabled elderly N64 mentioned, “I hope the government will take a priority to the elderly and show the equality on this special group, making us feel’we are the same’.”

#### Family doctor team

(1) Enlarging the scope of services and giving professional service (94/118)

The disabled elderly N3 mentioned that “I hope my family doctor can provide professional rehabilitation training for me at home.”

(2) Conducting regular health education for caregivers (88/118)

The disabled elderly N7 noted “I hope that family doctors can popularize hygiene knowledge, supervise family members and give them health guidance.”

(3) Establishing family hospital beds system (85/118)

The disabled elderly N76 mentioned, “I hope to have a hospital bed at home, and doctors visit regularly.”

(4) Calling more often with the contracted elderly to express concern (71/118)

The disabled elderly N6 mentioned, “I hope the family doctor can contact us frequently and show more care for us, especially for our health”; N11 said, “I hope family doctors focus more on the family members of the disabled elderly and help them release their stress.”

(5) Establishing the “green channel” (71/118)

The disabled elderly N67 mentioned, “I think CHS centers should open a “green channel” for the elderly. I queued for a long time when I got a vaccine last time, but my body couldn’t bear it.”

#### Family member

(1) Providing financial support (111/118)

The disabled elderly N54 mentioned that “children should observe filial piety to share the responsibilities and take care of the elderly.”

(2) Accompanying the elderly more and paying more attention to their psychological problems(109/118)

As the disabled elderly N72 mentioned, “It is good for my children to keep me company, I need them especially during the Spring Festival.”

(3) Looking after with more patience (87/118)

The disabled elderly N87 mentioned that “Family members mainly attend my daily life. So, I think that they need to be more careful and patient in the process of home care.”

(4) Learning health care knowledge (73/118)

The disabled elderly N53 said, “I hope that my family members take the initiative to attend the health lecture and learn health care knowledge.”

#### Society

(1) Providing voluntary services (106/118)

The disabled elderly N77 mentioned, “I wish that social organizations can exert their power and help more people with disabilities, for example, I need volunteers sometimes come to my home and give me a helping hand such as shopping.”

(2) Creating a harmonious social atmosphere and respecting the elderly (88/118)

The disabled elderly N67 mentioned that “A harmonious social atmosphere is necessary for the elderly, social group should consider more about the elderly and raise them the social status, which can make us realize the value of ourselves.”

(3) Establishing a professional health care service platform of home care (63/118)

The disabled elderly N67 mentioned that “Except for the elderly post, there is no formal and professional platform to provide home care assistance for the disabled elderly. However, the fact is that we can’t afford the expensive services of the post.” The N30 said, “Although my nanny does a good job in looking after me, she is less educated and lacks knowledge about nursing.”

(4) Strengthening assistance from neighborhood committees (56/118)

As the disabled elderly N64 mentioned, “Now, the neighborhood committees show little care to the elderly and committee’s members do not carry out their obligations. I hope that committees’ members really do some practical work.”

(5) Offering financial support (18/118)

The disabled elderly N51 mentioned, “I hope that social organizations can give more financial assistance or subsidized items to the disabled elderly, because they are shouldering the heavy burden.”

#### The disabled elderly

(1) Inability to undertake responsibility for health due to incapacity (89/118)

Most of the disabled elderly mentioned in the interview that they couldn’t bear health responsibilities anymore, even their consciousness would be blurred from time to time.

(2) Following the doctor's advice (22/118)

The disabled elderly N45 mentioned, “The family doctor’s advice is beneficial, so I have broken some old habits, such as smoking and drinking and live in a good lifestyle.”

(3) Controlling the temper (11/118)

The disabled elderly N76 said, “I have a bad temper, and I will be angry with some trifles, which has made my health conditions more terrible. Therefore, I must control my temper now.”

**Fig. 1:**
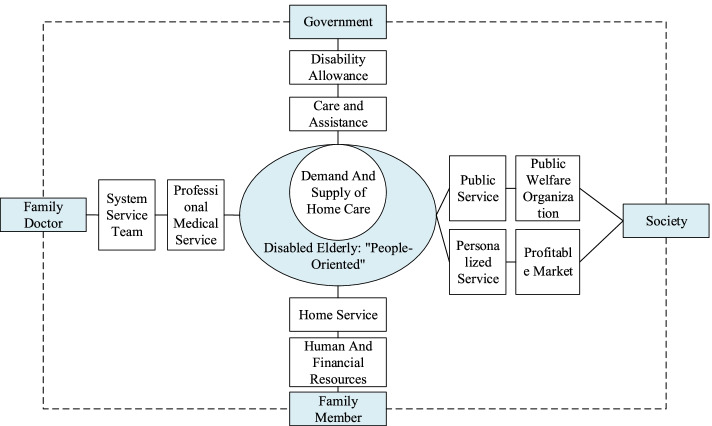
Schematic diagram of the relationships among multiple subjects which involved in the service supply process of home care for the disabled elderly. Note a: The dotted line represents that the subjects have not yet formed a close cooperative relationship

Based on the interview results, the researchers have drawn up a schematic diagram which describes the relationships among multiple subjects which involved in the service supply process of home care for the disabled elderly, as shown in Fig. 1.

## Discussion

### The necessity of multiple subjects participating in home care for the disabled elderly

At present, a global agreement is within reach that population aging has been a tough sell exerting a profound influence on the sound development. China is no exception [11]. Moreover, China’s aging population is characterized by aging, empty nesting, high disability rate and incidence of chronic diseases. It is more difficult to tackle the problem [12]. Affected by the Chinese traditional culture, the elderly is reluctant to leave their familiar living environment and go to the institutions of health care [13].

On the one hand, with the further increase of disabled elderly and the decrease of family size, home care provided by children and family members is becoming more and more inadequate [14]. On the other hand, welfare pluralism theory provides a theoretical basis for the multiple subjects to participate in the process of home care. The theory of welfare pluralism notes that through the interaction and coordination among the multiple subjects, care resources can be effectively integrated, so as to maximize welfare [15]. However, the results show that family members still play a key role in supporting the disabled elderly in China. The government takes main responsibility for social disadvantaged groups, family doctors may only play a guiding role, but social organizations play a very little compensation role. In addition, due to the increasing number and the long duration disability period of the disabled elderly, care resources provided by a single sector cannot tailor to the large demand of the disabled group. Therefore, it is necessary for the government, family members, family doctor team, society and other multiple subjects to participate in the home care system and form an integration mechanism in which multiple subjects share the responsibility. In a bid to wield the resources from relevant sectors to popularize home care, the cooperation among multiple subjects can complement each other’s advantages and provide home care resources more efficiently and accurately.

Some countries or cities that aged faster have relatively mature theoretical and practical exploration on the care of the disabled elderly. Denmark and Sweden put forward the theory of aging-in-place in 1960, emphasize that families should connect community resources and join together to build a care support network for the elderly. Through using community resources, the elderly can age naturally in their familiar places [16]. France proposes a government-led care service system for the disabled elderly. The role of government is handling social assistance initiated by the disabled elderly and arranging eligible elderly people to live in care institutions [17]. Australia encourages family doctors to provide door-to-door health services for disabled patients, with a view to effectively coordinating medical and health resources and improving the quality of lives of the disabled elderly [18]. Through promoting the development of community hospitals, Singapore provides care and rehabilitation for the disabled elderly. In addition, community workers arrange for the disabled elderly in poverty to welfare institutions [19]. With home care as the core concept, Taiwan launched the “Ten-Year Plan for Long-Term Care” in 2007, and explored the integrated care model around the core functions of primary medical services and family daily care [20]. In view of the above investigation and research, in order to meet the higher care needs of the disabled elderly, Beijing is shifting to the care model of community-based home care, through strengthening the multilateral coordination between the government, family doctors, family members, society, and the disabled elderly, different subjects could realize the effective linkage of elderly care service resources in the end.

### Inefficient coordination mechanism among multiple subjects in the process of home care for the disabled elderly

Although the government, family doctors, family members, society, and even the disabled elderly themselves, all perform various functions of home-based care services to varying degrees, the research findings indicate that the coordination mechanism among the subjects has not yet been established. After extensive review of relevant literature and repeated discussion by panel experts, this study has summarized the functions of multiple subjects in the home care of the disabled elderly, as shown in Table 5 below:

**Table 5:** The functions of multiple subjects in home care of the disabled elderly

Subject	Government	Family Doctor	Family Member	Society		The Disabled Elderly
				Non-profit, Organization	For-profit, Market	
Role	Supplier, Policymaker; Supervisor	Supplier	Supplier, Consumer, Supervisor	Supplier, Supervisor	Supplier	Supplier, Consumer, Supervisor
Motivation	Government, Responsibility, Public Interests	Duty, Responsibility	Responsibility, Family Interests	Spontaneity, Public, Interests	Profit	Independence, Health Rights
Aim	Promote Health Equity	Protect Health	Maximize Self-utility	Maximize Social Benefits	Maximize Benefits	Maximize Self-Health Rights
Mechanism	Bureaucracy	Job duty	Family Mutual Aid	Voluntary	Market	Autonomous Participation
Advantage	Authoritative Guidance	Professional Assistance	Emotional, Support	Free Supplement	Diversified Services	Proactive Cooperation
Disadvantage	Government Dysfunction	Low Responsibility	Weak Support	Voluntary Dysfunction	Market Dysfunction	Capability Limitations

#### Lack of consistency in the goals of multiple subjects

According to the Table 5, each subject has its own focus, and there is a lack of unified consensus about caring for the disabled elderly. Long-term elderly care services can help meet the most pressing needs of the disabled elderly and improve their quality of life, which are provided by their family members on the basis of blood relationship and ethics. While family doctors, motivated by their duties and job requirements, provide medical care and medical insurance for all patients within their personal responsibilities [21]. As the final target of the for-profit market is pursuing maximum benefits, quality, and types of care services for the disabled elderly can be improved through competition under such circumstances. Until recently, an increasing sense of gain and well-being for seniors with disabilities has been regarded as one of the government’s medium-and long-term plans. However, the harsh terms of assistance and low subsidies are far from meeting the general needs of the disabled elderly [22]. Moreover, the disabled elderly with self-awareness hope to meet their personal needs and maintain their personal health, which reflects the interest appeals for multiple subjects from different sides.

#### Low participation and insufficient resources input of multiple subjects

A multi-subject coordination mechanism involving home-based care for the disabled elderly has initially taken shape, but the existing service forms are merely the accumulation of functions among various subjects. Family members continue to be the primary source of home care. Furthermore, the interview results show that a lack of participation by multiple subjects in home care results in a low level of service supply, and the total resources input cannot meet the demands of the disabled elderly. As a result, families are forced to take on responsibilities that are beyond their capabilities [23]. Moreover, it is difficult for the government to consider the needs of each disabled elderly due to limited resources and difficulties in equal allocation. In addition, the scale and number of employees also hinder family doctors from serving the disabled elderly better. Furthermore, public welfare social organizations are rarely involved in home care because they lack sufficient motivation [24], whereas the limited purchasing power of the disabled elderly in the for-profit market results in a long payback period, which limits the development of service forms, standards, and even the entire industry [21]. This indicates that social resources and power have been underutilized. Therefore, the above problems reside in the fact that multiple subjects are neither fully involved in home care of the disabled elderly nor properly motivated to provide sufficient resources for those disabled people.

#### Lack of coordination among multiple subjects involved in the process of home care

It is obvious that actions of multiple subjects involved in the process of home care lack coordination, and the home care resources provided for the disabled elderly by the government, family doctors, family members, and society are not well-coordinated [25]. With the increasingly serious aging of Chinese society, the disabled elderly and their families are suffering more from triple stresses of the finance, body and psychology. They are still the primary undertakers of home care. As a result, the government bears the responsibility of providing care and assistance to the disabled through Care Payment or other means to alleviate their financial burden. Family doctors should play an important role in the health management and medical care of the disabled elderly. Public welfare social organizations take the initiative to provide the voluntary services, and the profitable market triggers a cycle of virtuous competition [26]. In this way, multiple subjects can provide certain supplementary services through their own platforms, thus reducing the burden of the disabled elderly and their families. However, there are differences in the roles and responsibilities among these different subjects, which makes it difficult to coordinate and integrate the resources of each subject, and causes the poor quality of home care for the elderly with disabilities.

### The diversified care services supplied by multiple subjects to satisfy various demands of the disabled elderly

#### Government guidance

##### Improve the subsidy policy and guarantee the disabled elderly’s legitimate rights and interests

In 2019, the Beijing Municipal Civil Affairs Bureau formulated the policy of “Administrative Measures for the Administration of Subsidies for Elderly Care Services in Beijing”, which assesses the eligibility of the disabled elderly who have submitted the application for subsidies. Then, in light of different degree of disability of the elderly, the government will provide nursing subsidies ranging from 200 to 600 yuan for them [27]. However, many disabled elderly families can hardly pay for their expenses. Taking care of the disabled elderly at home makes things worse, and government subsidies are just a drop in the bucket. Most disabled elderly families live in poverty and suffering. Therefore, the government should dynamically adjust the social welfare subsidy policy in combination with the regional economic development level and CPI rising index, give full play to the functional role of the financial department in social security reform, and steadily improve the social security level of the disabled elderly people [28].

The subsidy for disabled elderly people is issued by the government to their Incapacitated Security Card, a personal account that can only be consumed at certain service stations such as the elderly-care post, and this kind of allowance is not available for withdrawal. Consumer services provided by the elderly post include helping the elderly take a bath, cleaning their rooms, and changing the urine pads and so forth. Although the government has made great efforts, the effect of subsidy policy is less than satisfactory. Unreasonable consumption pattern and imperfect service model hinder those elderly from enjoying their legitimate rights, unaffordable price and poor quality of services in senior stations contribute to the unhealthy tendency in Chinese society. Therefore, government departments should improve the social recognition of the subsidies for the disabled elderly, strengthen the dynamic supervision of elderly care stations in society, and evaluate the service qualifications of third-party organizations, especially the service price and quality. Moreover, the government should establish a reasonable and effective supervision and feedback mechanism to ensure that the disabled elderly and their families can enjoy services without being restricted by location [29].

##### Enhance the policy publicity and ensure the disabled elderly to enjoy their benefits timely

One of the criteria for evaluating the effectiveness of the government’s efforts is the strength of policy publicity. Therefore, the propaganda department should enhance the popularization of the policy, and ensure the disabled elderly and their families can have access to the policy in time, especially for those living in impoverished areas. Local neighborhood committees should become the bridge for information transmission, connecting the government and the elderly disabled families. Council members in the committee should undertake their responsibilities for its management and policy publicity in local regions. Besides, the Civil Affairs Department should implement relevant public welfare policies and accelerate assessing the qualification of the disabled elderly to ensure that the policies for the elderly rights are fully implemented.

##### Promote the development of palliative care and speed up the legislation construction on euthanasia

Compared with other developed countries, the work of palliative care in China began relatively late. In July 1988, Tianjin Medical College established the first hospice care institution in mainland China, namely the “Hospice Care Research Center of Tianjin Medical College”. Since then, palliative care institutions have sprung up across the country, but most of them are concentrated in metropolises such as Beijing and Shanghai. Owing to the unreasonable distribution of medical and health resources, it is difficult to meet the general needs of patients for palliative care.

Palliative care includes multi-level service, aiming at alleviating the pain of patients at the end of their days and improve the quality of life [30]. However, influenced by the traditional Chinese culture, there is a general dispute about palliative care that whether patients should give up treatment. Ancient Chinese regarded hair as life, just as the saying goes, “Skin and hair are given by the parents, and they dare not hurt them.” While palliative care is a treatment to relieve, rather than cure. Restricted by their traditional ideas, the Chinese people ignore the advantages of medical services and the importance of death education [31].

Along with palliative care, China has also proposed the legalization of euthanasia since 1993, but it has not been put into practice so far [32]. The focus of this proposal is to give the suffered a right to choose death with dignity and bring the tortured an end when they can’t bear the pain of illness. As there are many diseases that can’t be cured by medicine, and there is still a long way to go in medical technology. Those critical patients suffered from both physical and emotional pains, so some of them may choose death with dignity. Therefore, it is urgent for the government to promote the development of palliative care and the legalization of euthanasia, and to ensure that dying patients such as some disabled elderly can make their own choices.

#### Family doctor assistance

##### Strengthen contract spirit and implement health management of the disabled elderly

More than 4,100 teams of family doctor have been established in Beijing. By 2019, 7.4 million people have signed up with family doctors, with an average of nearly 1,800 people in each team. Among the residents who have signed the contract, the signing rate of key group such as elderly patients with chronic diseases reached over 90. The National Health Commission made an announcement about family doctor service in 2019, which ask family doctors to provide door-to-door medical and health services for disabled and semi-disabled elderly, disabled people, terminally ill patients and other people who are in urgent need and extend the contracted services from institutions to communities and families [33].

Disabled elderly people, as a key group, are in urgent need of family doctor services. The community health service institutions located in Beijing all have started signing up services for disabled elderly people. About 4% to 5% of the contracted clients of each doctor team are disabled elderly people. In this study, 2 of the 118 disabled elderly people mentioned that they had signed with their family doctors but had never received any services, which indicated the contract between family doctor and the patient is invalid. Low contact frequency and trust may cause a bad contract service relationship. Currently, the effects of the family doctor project are not obvious. Therefore, family doctors should deliver health education to their disabled clients and make them realize the advantages of having a family doctor. Through long-term health education, family doctors can establish a stable relationship with their clients, and the elderly themselves can also enjoy the benefit from such contracted services [34]. At the same time, family doctors should actively connect with their clients, spread their services, increase the frequency of regular home calls, detect the health problems of the disabled elderly [35], and provide health management in time [33]. Only in this way can the family doctor team bridge the gap between the doctors and the disabled elderly and make their contract services more effective.

##### Expand the aspects of services to satisfy the diversified demands of the disabled elderly

Unlike healthy elderly, the disabled elderly have greater demands for family doctor services, particularly for family care bed. Currently, the CHS centers in Beijing provide limited medical resources for the disabled elderly, and the contract services are poor, which limits the benefit of those in need. For one thing, with limited family care beds, it is difficult for those disabled elderly to fully enjoy home-based injections, infusion care and other services; for another, they also face many difficulties when going to the hospital for medical treatment due to their bad physical situations. Consequently, the disabled elderly have great demands for family doctors to bring indoor medical services, such as injection and infusion.

In addition, studies have shown that early intervention of disability has a significant effect on controlling the disease process and improving health conditions [28], and the elderly with mild disabilities have a great demand for rehabilitation training in this research. However, the family doctor team lacks rehabilitation training services at present and the team doesn’t have any rehabilitation physiotherapist to meet the diverse needs of the disabled elderly in community.

Furthermore, staying at home for a long time can lead to a sharp decline in social relations of the disabled elderly, and they may be grumpy or depressed; their family members also have psychological burdens after long-term care [35]. In this study, the disabled elderly often consider that they are worthless and no longer able to contribute to society or even the family. They think that there are many drastic changes in their lives, which will bring great difficulties to their families. Therefore, it’s necessary for family doctors to expand contacted services, pay more attention to the mental health of the disabled elderly and their families, and provide them with more medical assistance and psychological counseling, so as to improve the life quality of the disabled elderly.

#### Family member guardianship

“Getting old before getting rich” is the mainstream trend in China’s aging society [36]. As a developing country, China cannot completely assume the responsibility of elderly care. Therefore, home care is still the basic care mode for the elderly [37]. The backbone of elderly care continues to be the young people who provide care and support.

##### Clarify the division of responsibilities within the disabled elderly’s family

The results show that in most disabled elderly families, spouses are the main caregivers, accounting for 35.6% of the caregiving role. They gradually replace their offspring to become a major caregiver of the disabled elderly [38], which is in line with studies on the topic. Most of the families interviewed have two children, which is not affected by China’s one-child policy (1980–2016). It can be said that family elderly care is guaranteed, but the fact is that children in families of the disabled elderly are shouldering less and less responsibility. Generally, those children have their own family and they are busy with work, which makes the sharing of family responsibilities unbalanced, and further causes one of the dilemmas for the disabled elderly [38].

There is a deep-rooted idea of “raising a son as a guarantee against old age” in China, which comes from Chinese traditional filial piety culture [39]. Most elderly people with son and daughter said in the interview that they would choose to live with their son. A common phenomenon is that children take care of their disabled parents in turns. When the core value of filial piety culture returns to kinship, it weakens the binding force of children’s support for their parents [40]. As a result, in some families, children have great disputes over pension and daily care for their parents and even shirk their responsibilities.

Therefore, when it comes to supporting parents, the offspring in multiple-child families should be fair and just, and distribute responsibilities according to income and residence distance to the elderly, particularly in the distribution of pension and medical expenses [38]. These measures can prevent children from disharmony and protect one of them from undertaking too much pressure due to improper arrangements. What’s more, the eldest son or daughter should play a leading role, coordinate family relationship, strive to create a positive family atmosphere and make sure the disabled elderly live in harmony.

##### Provide financial support, high-quality care and company

Family care usually contains three aspects: material support, daily care, and spiritual comfort. Family members, as the guardian of the disabled elderly, are the first to bear the disabled elderly’s healthcare expenditure. They should provide financial support to meet the material needs of the disabled elderly, and protect health benefits for the elderly by purchasing movable beds, installing anti-slip rails, and other equipment suitable for them [29].

Family caregivers play an important role in the home care for the disabled elderly, and their knowledge affects the quality of home care [41]. As primary caregivers, family members should take initiative to learn health knowledge and actively participate in the care training organized by the contracted family doctor, so as to provide high-quality care for the disabled elderly at home. At the same time, they should keep in touch with the doctor to exchange the health condition of the elderly and ensure timely medical treatment if the elderly get sick.

In addition, there are many traditional festivals in China that symbolize family reunions, such as the Spring Festival, Mid-Autumn Festival and Double Ninth Festival [42]. The Chinese people pay more attention to family reunion, and the long days of vacation give people a chance to stay with family. In this study, the disabled elderly have extremely high expectations for companionship, as 109 out of the 118 interviewees expressed that they wished to live with their families during traditional festivals. But the fact is that many children have little time to accompany the disabled elderly, except the family caregiver. Therefore, family members should actively coordinate their personal time, pay attention to creating a good family atmosphere during traditional festivals, and provide care and companionship to the disabled elderly. In daily life, they should also focus on the psychological problems of the elderly and give timely assistance to reduce geriatric depression [43].

#### Society participation

##### Create a good social atmosphere to respect and care for the elderly

The current society has some uncivilized concepts and behaviors in the treatment of vulnerable groups such as the disabled elderly, which require great efforts to change. Related studies have shown that social support can relieve loneliness and depression [44]. The research has shown that one of the obvious psychological problems of the disabled elderly is that they consider themselves a drag on their families, a burden on society, and deny their value in life. When they feel discriminated against by others, they worry more about their old age. Therefore, it is important to enhance the cultivation of personal morality and social support, care for the physical and mental health of the elderly, improve the civilization of the whole society, and guide the people to practice the core socialist values [23].

##### Promote voluntary and build a community service system

Facing the dual dilemma of insufficient care services provided by home caregivers and the government, community voluntary become a supplemental resource [44]. The results show that Beijing has not yet formed a systematic volunteer service system for the disabled elderly groups, who are in urgent need of services including accompanying the elderly to the local hospital, providing temporary care at home, purchasing daily necessities, etc. Therefore, community volunteers have great potential to ease the pressure on home care services of the primary caregivers.

First, long-term and stable interpersonal relationships make it easier for the disabled elderly and their families to release pressure and seek solace from the volunteers [45, 46]. In this study, most disabled elderly and their families hope to have a volunteer to help them. While the current volunteer activities in Beijing are carried out by some government institutions or staff, unable to provide sufficient funds and long-term voluntary service.

According to relevant studies, community activities, group participation, and friendship networks are beneficial to the elderly’s health condition [47]. Therefore, it is recommended that neighborhood committees and other social institutions actively mobilize party members or other volunteers in the community to establish a one-on-one assistance mechanism with the disabled elderly, so as to provide psychological care and support, and assist disabled elderly people to receive medications from hospitals and accompany them to receive medical treatment or other services.

Second, volunteers should be given systematic and standardized training on healthcare knowledge, and the neighborhood committees should match volunteers to the elderly who need their help the most effectively. Volunteers who have received training can help family members with basic emergency treatments when they are faced with sudden health troubles of the disabled elderly, so as to protect their lives [23].

Third, establish an incentive mechanism for volunteers. “Time Bank” or “Public Welfare Bank” may become an effective method to improve motivation [16]. That means, every registered volunteer and their service time, as well as the types of service they provided, will be recorded in the digital file. Volunteers get credit when they help the disabled elderly, from home care to medical care. These credits can be saved in the “Time Bank” and spent when volunteers are in need. The elderly can also participate in volunteer activities. Research shows that the disabled may be a very important contributor of volunteer organizations, especially middle-aged and those with the potential to generate capital in social networks [46]. Such schemes can encourage volunteers to help the disabled elderly, make their services credit a heritage that can be passed to next generation. In addition, it can ensure sustainable voluntary services, give full play to the functions in social support and cultivate a good social atmosphere of mutual help.

##### Improve market environment of home care and attract more enterprises to invest the elderly market

There are still many thorny problems in the service market for the disabled elderly. The families interviewed in this research have mentioned that they found it difficult to obtain reassuring services in the market when they need to hire nannies or enjoy other care services [19].

In 2016, in order to further stimulate market vitality and encourage social capital to enter this industry, the General Office of the State Council promulgated the “Several Opinions on Fully Liberating the Elderly Care Service Market and Improving the Quality of Elderly Care Services”, which provided loans for relaxing the access conditions of the senior care service industry. In 2019, the General Office put forward the “Opinions on Promoting the Development of Elderly Care Services”, in order to remove the obstacles to the development of the elderly care services industry and carried out all-round work arrangements to eliminate obstacles to the elderly care services industry. However, the current market development of the home care services industry for the disabled is still sluggish.

Therefore, society should promote the concept of respecting and loving the elderly, attract more enterprises to invest in the elderly care services market, establish a comprehensive regulatory system to encourage benign competition and improve the market climate of home care services. In this way, on the one hand, it can meet the demands of the disabled elderly and their families, thus liberating family labor [47]. On the other hand, a competitive care services market will help companies keep improving and provide better services in home care [48].

#### The disabled elderly cooperation

Traditional health theory suggests that when health hazards are related to the individual lifestyles and behaviors, and medicine don’t work, then the individual’s control of their own behavior will become an important way to promote health [49]. In other words, when an individual chooses an active and healthy lifestyle, they tend to have better health conditions and pay less for medical services. For example, abandoning unhealthy behaviors such as smoking and alcohol abuse would keep patients away from diseases. At the same time, related research shows that there are two arguments supporting the elderly to be responsible for their own health: one is for their own interests and the other is relief on social pressure [50]. That means, first, the elderly should protect their own health; second, they should reduce the medical burden of the society.

In terms of health, personal responsibility means reducing health risk factors to a level that individuals can reasonably control, and the fulfillment of personal health responsibility is mainly manifested in the management of one's own health and self-education [51], but there are limitations in personal health management for the elderly with disabilities. The study demonstrates that most of the disabled elderly, especially those in bad situations, have lost the ability to manage their own health. They are bedridden for a long time, unable to defecate or eat on their own, and some of them even have lost their consciousness. Therefore, it is difficult for the disabled elderly group to become the first person responsible for their personal health, and it is impossible to be too strict with them. Other subjects involved in home care cannot rely too much on the disabled elderly’s self-care, whereby “self-care” is potentially unachievable and becomes care left undone. This will create a culture of blame for self-care, attributing the elderly’s poor health to their personal behavior [52]. This is obviously unreasonable for the disabled elderly to become the first person responsible for health. When the disabled elderly are still partially capable of acting, they should take initiative to protect personal health; when they are still conscious, they can actively adjust their personal emotions, seek assistance from others in time, and maintain their own mental health.

### Advice on improving the coordination mechanism of multiple subjects involved in home care for the disabled elderly

**Fig. 2:**
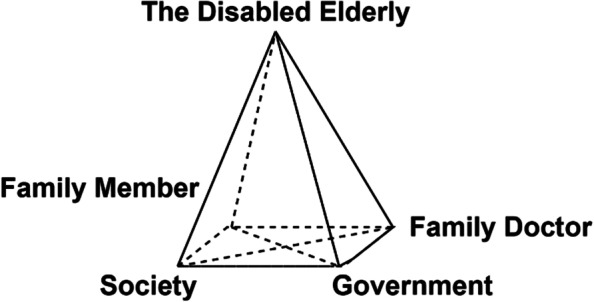
A collaborative framework of multiple subjects involved in home care for the disabled elderly

After an extensive review of relevant literature and discussion by panel experts, this study summarized a collaborative framework of multiple subjects involved in home care of the disabled elderly, as illustrated in Fig. 2. The disabled elderly as the vertex is located at the top of the quadrilateral cone, and the other four subjects serve as the endpoints of the bottom quadrilateral. Each subject as a core element, is linked by other four subjects and forms a multi-subject cooperation mechanism. From this quadrilateral cone cooperation mechanism, communication, collaboration, and resources exchange can be realized among multiple subjects. Each subject establishes a close relationship with others, the government formulates welfare policies for disabled elderly and their families, as well as promotes incentive policies for family doctors to encourage them to help the disabled elderly and gives policy guidance to social organizations. Other subjects in this cooperation mechanism give feedback to the government at the same time. Family doctors provide medical services for the disabled elderly, give professional technical guidance for the family members of the disabled elderly and they can also join the social organizations as volunteers, which is a useful supplement to keep this mechanism work effectively. Family members of the disabled elderly provide daily care for the disabled elderly, connect family doctor to report the health conditions of the disabled elderly and sometimes ask social organizations for help. Society plays a supporting role in providing help to family doctors, the disabled elderly and their families. Therefore, in order to establish a multi-subject quadrilateral cone cooperation mechanism for the disabled elderly people in Beijing, the following suggestions are presented.

#### Strengthen the integration of internal resources within multiple subjects

The mismatch between the resources available to an institution and the services it provides is often due to institutional structure, information asymmetry, or other reasons. Therefore, there is an urgent need for concerning subjects to integrate their internal resources from different aspects.

Policies related to elderly care are fragmented and implemented by different government departments. For example, the civil department is only in charge of care subsidies for the disabled elderly, and the medical security department only is responsible for the medical insurance reimbursement ratio. Under this management system, government is less efficient in distributing resources and there is a large gap between the services that are provided and the needs of the disabled elderly. This results in the elderly with disabilities being indivisible individuals, but their demands are scattered. A clear policy and action plan can help reduce inefficiency and division in the health care system. Therefore, the government should promote coordination and information exchange among departments [52] and establish a better management system to realize resources integration.

To integrate family doctor team resources, we should flexibly and dynamically adjust the composition of service personnel and provide tailor-made services to the disabled elderly and their families to meet the diverse needs of the disabled people [53].

To integrate idle human resources and finance is the main problem faced by family members It is necessary for families to allocate human resources and financial resources reasonably so that the disabled elderly can obtain the best home care service at the lowest cost [52].

Social resources integration requires information technology to sort out and match the demands of the disabled elderly and the care services they need.

#### Define the position of the responsibilities for the disabled elderly and promote the resources integration among multiple subjects

Responsibilities allocation and information sharing are prerequisites for multiple subjects to cooperate with each other and provide services for the disabled elderly in the process of home care.

First, the family and the disabled elderly themselves have a fundamental responsibility for the home care services [54]. Traditionally, individuals are mainly responsible for their own health, and family members are the most natural and stable caregivers. However, there are many factors that affect the quality of home care for the disabled elderly, such as the change in family size, the duration of care extends, etc. These all increase the cost and difficulty of home-based care for disabled elderly [53]. To maintain the basic roles of the family, the government should provide economic subsidies and policy support, and family doctors should offer technical guidance and health education to improve family members’ care. Social welfare organizations should reduce the pressure on those caregivers through sufficient assistance. At the same time, paid alternative care services should also be developed so that families can have temporary rest by purchasing alternative services.

Second, the government is the last line of defense for the home care security system. Therefore, the government should introduce a series of measures to strengthen assistance for those vulnerable disabled elderly, pay more attention to solving the problems caused by the elderly and their families, and make sure enough living and medical resources are accessible to everyone. Encouraging family doctors and social groups to participate in elderly care services for the disabled is also an important step for the government. This will be explained in detail as the following incentive policies for multiple subjects.

Third, the functions of family physician teams and other social groups are complementary [26]. Family doctor team can be a useful supplement to home care services, and by contracting services and health management, they can effectively extend the life span of the disabled elderly and improve their quality of life. Public welfare social organizations can bridge the gap when other subjects are unable to provide care services, and they will provide social support for the disabled elderly, and build a good mutual assistance system. Through competition, the for-profit market also can provide affordable and high-quality services for disabled elderly people.

#### Incentive policies for multiple subjects involved in home care services for the disabled elderly

In order to promote the collaboration of multiple subjects, it is necessary to establish incentive policies for them to take initiative to provide home care services. The government should take responsibilities in the care of the disabled elderly as they are vulnerable group. It is an important responsibility of the government to mobilize the active participation of multiple subjects [21]. For example, the government can give some subsidies or even weekly vacations to the caregivers in the families of the elderly with disabilities. In addition, targeted funding can be set up in the family doctor team to encourage doctors to provide high-quality care services to the elderly with disabilities. Moreover, the government can promote non-profit social welfare organizations to expand their social benefits and support the for-profit market with public facilities and uniform staff training.

## Conclusions

This study highlights that home care resources only provided by a single subject cannot meet the diverse needs of the disabled elderly, which may lead to inefficiencies and disconnections of the supply chain of resources. Therefore, this study proposes a new mechanism to understand and analyzes the problem of home care for the disabled elderly. On the one hand, the multi-subject collaboration mechanism clarifies the responsibilities of each subject and analyzes the ranking of responsibility-sharing among multiple subjects, so as to solve the limitations of past studies in addressing the care needs of disabled elderly people only from a single subject. On the other hand, this study explores the effective linkage among various subjects by establishing partnerships and achieving the coordination of service provision and home care resources supplement, which solved the problems of fragmentation of home care services among multiple subjects in past studies. In addition, this study emphasizes the responsibility of the disabled elderly, whereas past studies have tended to treat the disabled elderly as passive recipients of home care services, which is not conducive to their physical and mental health.

Home care for the disabled elderly is becoming more intertwined with society. The demands of the disabled elderly are becoming increasingly diversified, and the participation of multiple subjects in the process of home care is becoming more and more important. However, there is currently insufficient home care provided by multiple subjects, and there is a lack of a coordinated mechanism for multiple entities to support the disabled elderly. Therefore, multiple subjects in Beijing should integrate care resources to provide care services for the disabled elderly more efficiently and precisely (e.g., the government should increase the inclination of relevant elderly care policies and strengthen publicity and guidance; family doctors should improve service quality; citizens should create a good atmosphere for the elderly; actively participate in the service industry for the disabled elderly, etc.), and thus improve the quality of lives of the disabled elderly and enhance their sense of well-being. Eventually, the ideal goal of making every elderly with disabilities have a sense of security, support and happiness will be realized in the near future.

Although the research included the disabled elderly in both urban and suburban and try to build the multi-subject cooperation mechanism of home care for the disabled elderly, it has limitations. It is an exploratory study, only a subset of the disabled elderly in 4 districts selected from 16 districts in Beijing were interviewed. The representativeness of the study was relatively limited because the interviewees are limited by the scope of their personal cognition and the population of small towns or rural areas may have a different attitude from that of a large Beijing agglomeration. Therefore, the results of our research cannot be applied to the entire Chinese population, it is worth considering conducting similar studies in smaller centers throughout the country in the future, which will allow a better understanding of the situation throughout China.

## Data Availability

Transcripts will not be shared for online access to protect the anonymity of the participants. Readers who wish to gain access to the data can write to the corresponding author.
